# Proteomic composition of the acrostyle: Novel approaches to identify cuticular proteins involved in virus–insect interactions

**DOI:** 10.1111/1744-7917.12469

**Published:** 2017-06-22

**Authors:** Craig Graham Webster, Mäelle Thillier, Elodie Pirolles, Bastien Cayrol, Stéphane Blanc, Marilyne Uzest

**Affiliations:** ^1^ INRA, UMR 0385 BGPI CIRAD‐INRA‐Montpellier SupAgro Campus International de Baillarguet Montpellier Cedex France

**Keywords:** acrostyle, aphid, cuticular protein, immunolabeling, peptide array, stylet

## Abstract

The acrostyle is a distinct anatomical region present on the cuticle at the inner face of the common food/salivary canal at the tip of aphid maxillary stylets. This conserved structure is of particular interest as it harbors the protein receptors of at least 1 plant virus, *Cauliflower mosaic virus*, and presumably has other roles in plant**–**insect interactions. Previously we reported immunolabeling of a highly conserved motif of cuticular proteins from the CPR family (named for the presence of a Rebers and Riddiford consensus) within the acrostyle. Here we report the development of novel tools to further study the proteomic composition of this region and to identify proteins involved in insect**‐**virus interactions. Using a series of antibodies against cuticular proteins from the RR‐2 subfamily, we identified additional peptides present within the acrostyle. Our results demonstrated that the acrostyle is a complex structure containing multiple domains of cuticular proteins accessible for interaction. In addition, an array of overlapping peptides, which covers the diversity of the majority of the RR‐2 subfamily, was developed as a generic tool to characterize cuticular protein/pathogen interactions. Upon probing this array with *Cucumber mosaic virus* particles, consensus peptide sequences from hybridizing peptides were identified. Use of these novel tools has extended our knowledge of the proteomic composition of insect maxillary stylets and identified sequences that could be involved in virus binding, thus contributing to further elucidation of the various properties and functions of the acrostyle.

## Introduction

Aphids are notorious in agriculture for the damage they cause to a wide variety of plants, including many important crops (Dixon, 1998; Dedryver *et al*., 2010). Such damage manifests mainly as: (1) consumption of large amounts of sap, leading to reduced vigor and stunting of colonized plants, which is exacerbated by the aphids ability to reproduce rapidly parthenogenetically (Tagu *et al*., 2008); (2) aphid honeydew excretions containing a high concentration of sugar that favor the development of fungi (such as sooty molds), which may also impact host plant fitness; and (3) the saliva of a few aphid species also displays toxic properties (Nicholson *et al*., 2012). Beyond these direct effects however, indirect damage due to the highly efficient transmission of hundreds of virus species (Hogenhout *et al*., 2008) is by far the most severe and detrimental consequence of aphids infestation, resulting in reduced host fitness and huge losses in production quality and yields (Dedryver *et al*., 2010).

Two main modes of virus transmission by aphids (and by insects in general) are known to occur and are typically distinguished by the ability of viruses to persist within the aphid body (Hogenhout *et al*., 2008; Blanc & Michalakis, 2016). As the name suggests, persistent viruses (also referred to as circulative viruses), are retained by the aphid, following acquisition, for the remainder of its life. In this mode of transmission virions are internalized in the insect body. They ultimately reach the salivary glands, from where they may be transmitted through salivation while the insect are feeding on a plant (Brault *et al*., 2010). Noncirculative viruses, which represent the majority of aphid‐transmitted viruses, require much short periods of time for acquisition and transmission, usually in the order of seconds to minutes. These viruses are lost within several minutes of acquisition, or after aphid molting. They are retained only within the stylets of the aphid (Blanc *et al*., 2014).

The aphid stylet bundle consists of 2 pairs of stylets termed maxillary and mandibular, with the external mandibular stylets surrounding the inner maxillary stylets. The 2 maxillary stylets are structurally complex and form 2 canals, the food canal and the salivary canal, resulting from the interlocking of ridges and grooves on the edges of the stylets. At the distal end of the 2 maxillary stylets the food and salivary canals fuse into a common canal through which both aphid ejected saliva and ingested plant sap pass. This region therefore represents the location in the aphid stylet where virions can attach successfully during feeding on plant sap (or plant cell contents) and be released during salivation, and hence this region likely contains the binding sites of noncirculatively transmitted viruses (Martin *et al*., 1997; Powell, 2005; Uzest *et al*., 2007). Giving support to this theory is the presence of a distinct anatomical feature, the acrostyle, which is visible on the cuticle lining at the bottom of the common canal in each of the maxillary stylets (Uzest *et al*., 2010). P2 protein of the noncirculative virus *Cauliflower mosaic virus* (CaMV) binds directly to the acrostyle (Uzest *et al*., 2007). P2 is the helper component of CaMV that links the receptor(s) molecule(s) in the acrostyle and the virus particle in a very specific manner. P2 can be produced easily in artificial systems in a biologically active form and is therefore an ideal tool with which to characterize noncirculative virus receptors as well as the organs bearing such binding molecules (Uzest & Blanc, 2016). The acrostyle is conserved amongst at least the Aphidinae subfamily (Uzest *et al*., 2010) suggesting an essential function in aphid biology.

**Figure 1:**
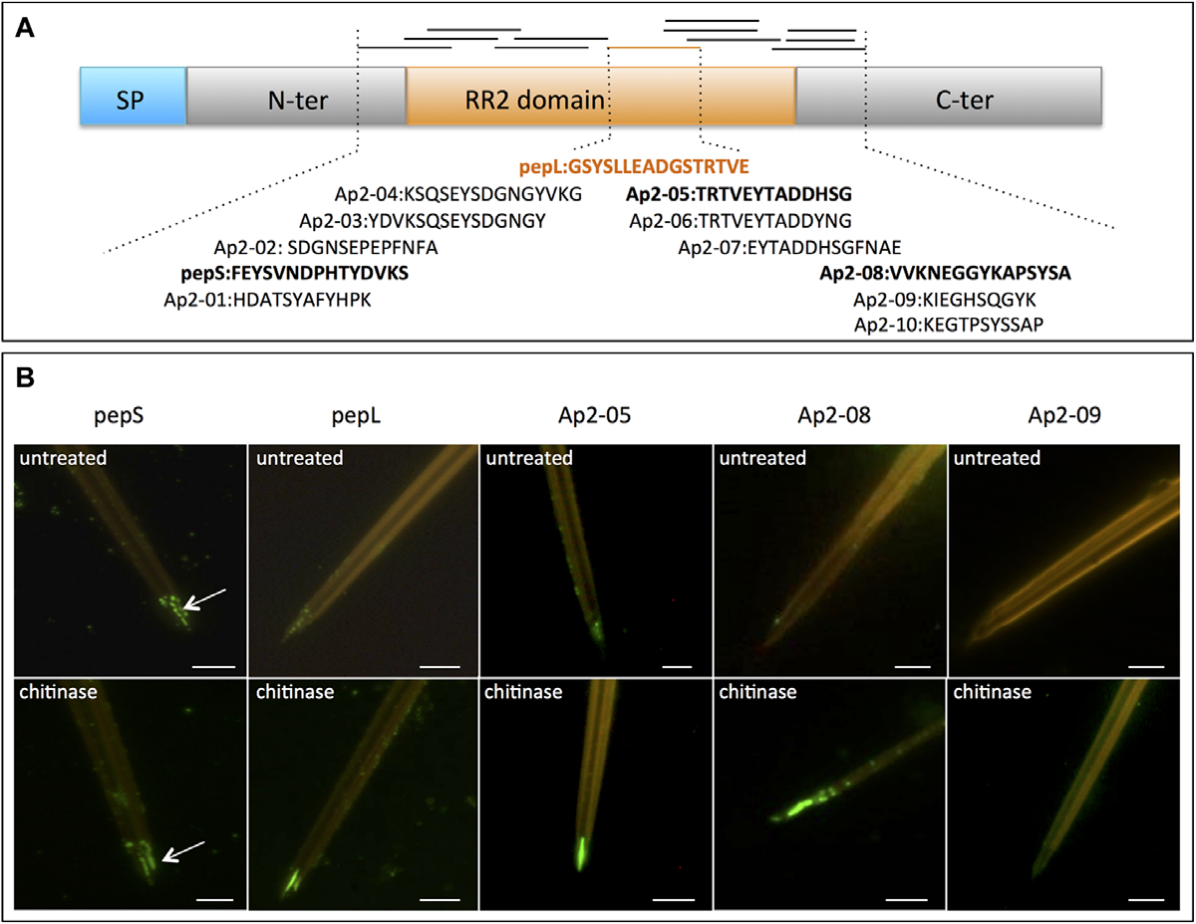
Mapping of accessible peptides of the RR‐2 chitin‐binding domain within the acrostyle of *Acyrthosiphon pisum* by immunolabeling with peptide‐derived antisera. (A) Schematic representation of the location of peptides used for antibodies production. A typical RR‐2 cuticular protein is represented, with the signal peptide (SP), and the N‐terminal and C‐terminal domains surrounding the central RR‐2 chitin‐binding domain. The peptides sequences (black and orange lines) are targeted upstream and downstream of the peptide pepL of the RR‐2 domain. Peptides likely to be accessible within the acrostyle identified by immunolabeling of dissected individualized stylets are in bold. (B) Immunolabeling of *A. pisum* stylets showing variety of labeling seen with stylets both undigested (untreated, top row) and digested for 15 min with chitinase (chitinase, bottom row). Scale bars of 5 μm are included. Immunolabeling is shown for anti‐pepS, anti‐pepL, anti‐Ap2‐ 05, anti‐Ap2‐08, and anti‐Ap2‐09 antibodies.

A 16**‐**amino**‐**acid motif, named pepL (GSYSLLEADGSTRTVE), is common in many cuticular proteins (CuP) from the RR‐2 subfamily [named for the presence of a motif described by Rebers and Riddiford (1988)], and is present within the acrostyle. However this motif is embedded within the chitin matrix of the stylet, and only poorly accessible to specific antibodies in the absence of a partial digestion of the chitin at the surface (Uzest *et al*., 2010). Typically RR‐2 subfamily proteins contain a central chitin‐binding domain (Andersen *et al*., 1995; Rebers & Willis, 2001; Willis, 2010) such as the conserved 64**‐**amino**‐**acids R&R domain (Cornman *et al*., 2008) in which the pepL motif is located. The R&R domain is surrounded by N‐terminal and C‐terminal sequences of variable length (Fig. 1A). In *Acyrthosiphon pisum* (Harris) these N‐ and C‐terminal domains are composed mainly of a variable number and arrangement of the amino acids proline, alanine, tyrosine and serine (Gallot *et al*., 2010).

The further characterization of CuPs and domains available at the stylet tip for binding plant viruses remains an important goal toward a better understanding of the transmission of noncirculative plant viruses. This characterization should also provide novel information on the physiological role of the acrostyle for the insect itself. Apart from the binding of plant viruses, the acrostyle is in contact with plant cell contents, phloem sap and aphid saliva. It might transiently retain compounds from the plant or from aphid saliva that are important for the aphid feeding process. For example, such compounds could be aphid effectors to counteract plant defenses that are delivered into plant tissues upon salivation (Jaouannet *et al*., 2014; Kaloshian & Walling, 2016; Mugford *et al*., 2016). Finding the partners of CuP domains that are accessible at the surface of the acrostyle should give new clues to deciphering plant**–**aphid interaction mechanisms. This knowledge is critical if we are to develop novel strategies for blocking receptors as a means of controlling viral transmission by aphids, or disturb aphid feeding. Methods for protein identification within stylets are urgently needed. However, stylets are small and their cuticle is a complex network of chitin fibers and heavily cross‐linked proteins, and rendering a successful extraction of pure CuP samples by classical proteomic approaches is very difficult. Therefore, in this work, we aimed to develop new tools to identify CuPs present in the acrostyle that can interact with viruses, aphid saliva or plant compounds. We produced a series of specific antibodies, which we determined that multiple regions of the core chitin‐binding domain of RR‐2 proteins are available close to, or at, the surface of the acrostyle. Additionally, we developed an array of peptides from the *A. pisum* RR‐2 subfamily and identified 2 conserved motifs interacting *in vitro* with *Cucumber mosaic virus* (CMV). CMV is a noncirculative viral species (unrelated to CaMV) retained in aphid stylets through direct binding of the coat protein (Gera *et al*., 1979; Chen *&* Francki, 1990) to receptors that are strongly suspected to be located in the common canal (Martin *et al*., 1997; Powell, 2005). These findings extend our knowledge of the CuPs of the acrostyle and suggest peptide motifs that may interact with noncirculative viruses and other compounds from plants and insects.

## Materials and methods

### Insect rearing

A colony of *A. pisum* (clone LL01) was maintained on Faba bean (*Vicia faba*) reared in an environmental growth chamber at a temperature of 23/18 °C and a photoperiod of 14/10 h (day/night).

### Antibodies

Peptides of 10**–**16 amino acids in length with sequences originating from various RR‐2 CuPs of *A. pisum* were synthesized. Antibodies were produced against sequences conserved across multiple RR‐2 proteins (e.g., Ap2‐04, pepL; Table 1); or against sequences that were more selective and present in only one or a few proteins (e.g., Ap2‐01, Ap2‐05; Table 1). The series of antibodies was designed to largely cover the diversity of CuPs but did not be exhaustively reflect of all possible sequences. The peptides were synthesized and used by Eurogentec (http://www.eurogentec.com) to immunize rabbits following standard protocols. Collection of derived antisera and subsequent purification on the peptide used for immunization were performed by the company. The antibodies anti‐pepL and anti‐pepS were produced as part of a previous study (Uzest *et al*., 2010). All primary antisera were used at a dilution of 1∶100 and 1∶1000 for stylet labeling and western blot/peptide array labeling, respectively, with either an Alexa Fluor 488‐ (GAR‐488; Molecular Probes) or an alkaline phosphatase (sc‐2007, Santa Cruz Biotechnology, Dallas, TX, USA) conjugated secondary antibody, also used at a dilution of 1∶400 and 1∶1500, respectively.

**Table 1:** Peptide sequences and the corresponding cuticular proteins for antibody production

			Labeling‡	
Antibody	Peptide sequence	RR‐2 proteins containing the exact peptide†	0 min	15 min
anti‐Ap2‐01	HDATSYAFYHPK	ACYPI000300, ACYPI004990	None (0/11)	None (0/11)
anti‐pepS§	FEYSVNDPHTYDVKS	ACYPI000983, ACYPI002200, ACYPI002889, ACYPI003453, ACYPI004074, ACYPI004893, ACYPI006045, ACYPI007329, ACYPI008534, ACYPIG166965, ACYPIG314262, ACYPIG566276	Dots (17/18)	Weak (12/12)
anti‐Ap2‐02	SDGNSEPEPFNFA	ACYPI000461, ACYPI002106, ACYPI006175	None (0/11)	Dots (6/21)
anti‐Ap2‐03	YDVKSQSEYSDGNGY	ACYPI000983, ACYPI002889, ACYPI004074, ACYPI004893	Dots (7/15)	Dots (3/10)
anti‐Ap2‐04	KSQSEYSDGNGYVKG	ACYPI000983, ACYPI001644, ACYPI002243, ACYPI002889, ACYPI003527, ACYPI004074, ACYPI004893, ACPYI56622	Dots (1/7)	None (0/25)
anti‐pepL§	GSYSLLEADGSTRTVE	ACYPI002200, ACYPI002889, ACYPI003453, ACYPI004074, ACYPI004113, ACYPI004983, ACYPI006045, ACYPI008534, ACYPIG166965, ACYPIG566276	Dots (17/21)	Strong (22/22)
anti‐Ap2‐05	TRTVEYTADDHSG	ACYPI004893	Dots (17/21)	Strong (21/22)
anti‐Ap2‐06	TRTVEYTADDYNG	ACYPI004074, ACYPI004113, ACPYPI006045, ACYPI007329, ACYPIG566276	None (0/10)	Dots (4/7)
anti‐Ap2‐07	EYTADDHSGFNAE	ACYPI000983, ACYPI008570, ACYPI56617, ACYPI56618	None (0/13)	Dots (2/17)
anti‐Ap2‐08	VVKNEGGYKAPSYSA	ACYPI002694, ACYPI004893, ACYPI006791, ACYPI007928 ACYPI008534, ACYPI009804	Dots (4/8)	Strong (22/24)
anti‐Ap2‐09	KIEGHSQGYK	ACYPI000983, ACYPI56617, ACYPI56618, ACYPIG435644	None (0/8)	None (0/11)
anti‐Ap2‐10	KEGTPSYSSAP	ACYPI001644, ACYPI002243, ACYPI003527, ACYPI56622	None (0/9)	None (0/12)

^†^ACYPI numbers of RR‐2 subfamily CuPs from *Acyrthosiphon pisum* containing a perfect match for the peptide sequence, names according to Gallot *et al*. (2010). Underlined ACYPI numbers represent incomplete genes that matched the corresponding peptide sequence.

^‡^ *In vitro* immunolabeling of *A. pisum* maxillary stylets without (0 min) or with 15 min of chitinase digestion treatment. Labeling at the acrostyle was rated either: none, incomplete (dots), weak, or strong.

^§^Previously developed (Uzest *et al*., 2010).

### Protein production

The P2 helper component protein of CaMV (Cabb B‐JI strain) was produced in the *Sf9* baculovirus insect cell system as a histidine tag fusion (6 histidines fused to the N‐terminus of the protein, hereafter HP2) and purified by affinity on a Ni‐nitrilotriacetic acid resin as described previously(Hébrard *et al*., 2001). Briefly, insect cells were harvested 48 h postinfection. Pellets were resuspended in DB5 buffer (50 mmol/L Hepes, pH 7.0/500 mmol/L LiSO_4_, 0.5 mmol/L EGTA and 0.2% w/v CHAPS) and were lysed by freeze‐thaw cycling at –20 °C. Cellular debris were removed by ultracentrifugation. The supernatant was then incubated with the resin. The HP2 protein was finally eluted from the resin using the DB5 buffer supplemented with 500 mmol/L of imidazole, which was removed by dialysis against DB5 buffer. Aliquots of 50 μL of purified protein were kept at **–**20 °C until use.

Virions of *Cucumber mosaic virus* (CMV) (Fny strain) were purified from zucchini (*Curcubita pepo* cv. Maraîchère; Vilmorin) at 14 d postinoculation (dpi) as previously described (Lot *et al*., 1972). Briefly, virions were extracted from infected leaves in 500 mmo/L sodium citrate (pH 6.5) with 40% chloroform and 0.04% w/v thioglycolic acid, clarified by centrifugation (8 850 r/m, 10 min, 4 °C) and precipitated with 10% w/v polyethelyne glycol‐6000. After resuspension in 50 mmol/L sodium citrate buffer pH 7.0, virions were finally precipitated by ultracentrifugation (160 000 × *g*, 2 h, 4 °C). Virus pellets were resupended in 20 mmol/L sodium citrate pH 7.0 and kept at **–**20 °C as a 50% glycerol stock until use. To obtain CMV coat protein (CMV‐CP), CMV particles were disrupted using 6 mol/L LiCl as described previously (Kaper, 1969; Lu *et al*., 2012). Disruption was confirmed by Transmission Electron Microscopy observation.

### Immunolabeling of stylets

Individuals of *A. pisum* were killed by freezing at **–**20 °C for 2 h prior to dissection. Stylets were dissected and individualized according to Uzest *et al*. (2007). Briefly, the stylet bundle was removed from the proboscis under a dissecting microscope (Leica MZ6), and the 4 stylets were then carefully separated using thin tweezers (N°5; Dumont) and insect pins (0.1 mm; Agar Scientifics, Stansted, United Kingdom). Subsequently, individualized stylets were transferred to siliconized cover slides and heated for 2 h at 50 °C. Individualized *A. pisum* stylets were immunolabeled according to Uzest *et al*. (2010). Briefly, stylets on coverslips were incubated in blocking solution (TS buffer [50 mmol/L Tris pH 7.4, 150 mmol/L NaCl] complemented with 5% skim milk powder) for 20 min at room temperature. They were then incubated with primary antibody at a 1 ∶ 100 dilution in blocking solution for 14 h, followed by secondary Alexa Fluor® 488‐conjugated antibody (GAR‐488, 1 ∶ 400 dilution; 4 h) with 3 rinses in TS buffer after each antibody. If no labeling was observed, digestion of chitin layers at the surface of dissected stylets was performed prior to immunolabeling as described in Uzest *et al*. (2007); this was done to determine if the epitopes recognized by the antibodies were present deeper in the stylet cuticle. Stylets were incubated for 15 min with 1 unit of chitinase from *Streptococcus griseus* (Sigma‐Aldrich, St. Louis, MO, USA). Coverslips were mounted on microscope slides and observed with an Olympus BX60 epifluorescence microscope.

### GST‐fusion production and evaluation of antibody specificity

pGEX peptide fusion plasmids were constructed using specific forward and reverse oligonucleotides (sequences listed in Table S1) including *Bam*HI and *Eco*RI restriction sites. Primers were annealed and then cloned in the plasmid pGEX‐3X (GE Healthcare Pittsburgh, PA, USA) plasmid cleaved with *Bam*HI and *Eco*RI enzymes. All constructions were verified by sequencing.

Glutathione‐S‐transferase (GST)‐peptide fusions were produced in *E. coli*, by induction of bacterial cultures at OD_600_ = 0.6 with 0.5 mmol/L IPTG for 3 h at 37 °C. Nonfused GST was used as a negative control. Cells were harvested, and resuspended in loading buffer (Laemmli, 1970), boiled for 5 min, and loaded on SDS‐PAGE gels. Samples were separated under denaturing conditions in 15% SDS‐PAGE, and blotted onto a nitrocellulose membrane (88018, ThermoFisher Scientific, Waltham, MA, USA). After blocking in TBST buffer (TS buffer with 0.05% [v/v] Tween 20) supplemented with 5% (w/v) skimmed milk powder, membranes were incubated for 1 h at room temperature with primary antibodies (rabbit antipeptide antibodies) diluted 1∶1000, followed by 1 h at room temperature in goat‐anti rabbit IgG‐HRP (sc‐2030, Santa Cruz Biotechnology, Dallas, TX, USA) used as secondary antibodies diluted 1∶5000. Interactions were revealed using chemiluminescence according to the manufacturer's instructions (ThermoFisher Scientific, Waltham, MA, USA).

### Production and interaction on peptide arrays

**Figure 2:**
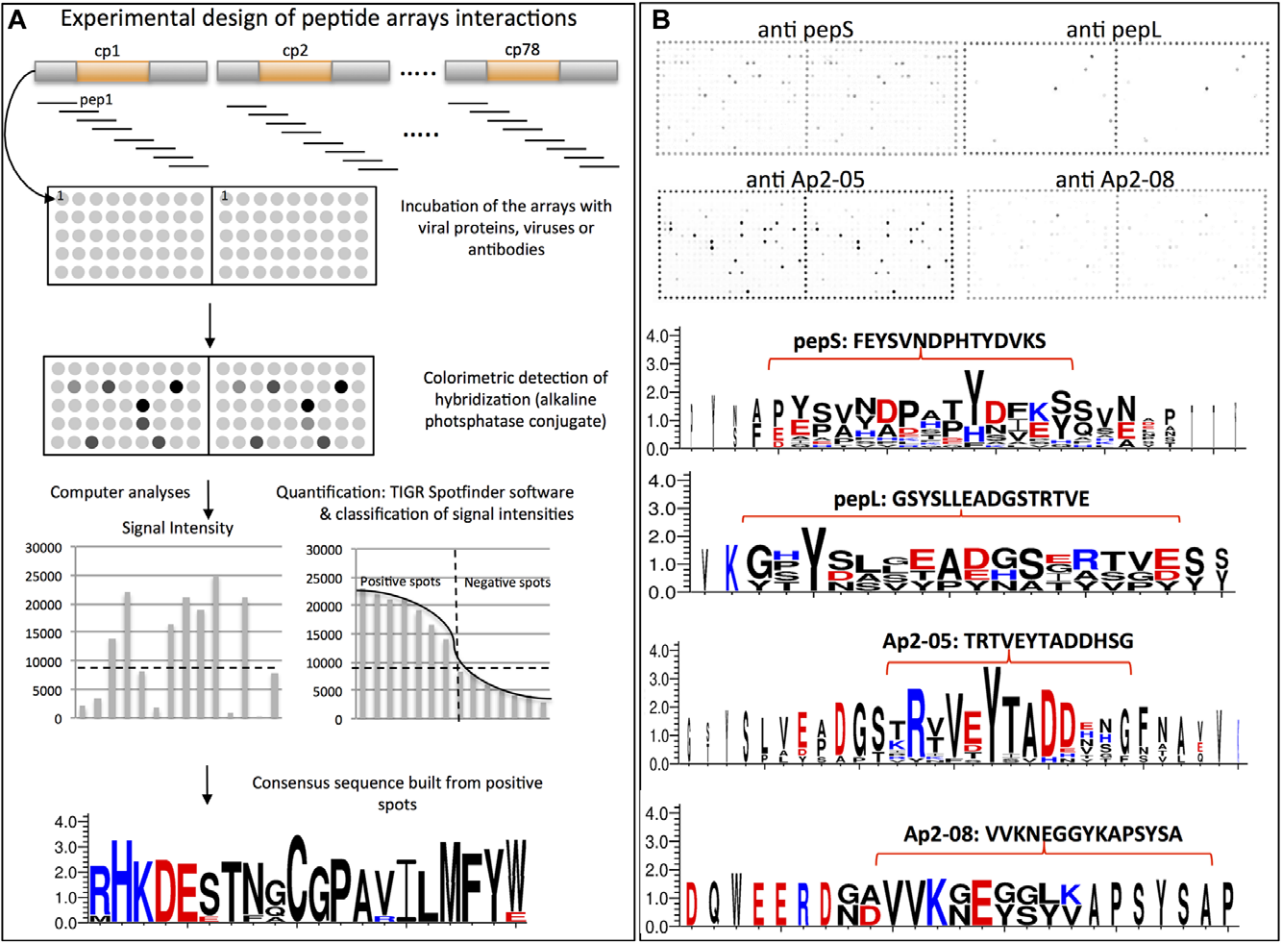
Interactions of antibodies on an array of peptides representing the diversity of RR‐2 proteins present in *Acyrthosiphon pisum*. (A) Overview of the design, hybridization, and analysis of peptide arrays. An array of 384 peptides, each 18 amino acids in length was designed from 62 complete RR‐2 subfamily proteins (from CuP1 to CuP78 according to Gallot *et al*., 2010, excluding the 16 incomplete protein sequences). Arrays were hybridized with either anticuticular protein antibodies or viral proteins and developed with colorimetric detection by alkaline phosphatase conjugated secondary antibodies. The level of hybridization was quantified (TIGR Spotfinder v3.2.1), the spots were then ranked by signal intensities to determine positive spots, and a consensus of interacting peptides produced (Weblogo 3, www.weblogo.threeplusone.com). (B) Results of interaction with specific CuP antibodies. Scanned arrays (top) hybridized with either anti‐pepS, anti‐pepL, anti‐Ap2‐05, or anti‐Ap2‐08, antibodies, or buffer as a negative control. Weblogo consensus sequences of interacting peptides (bottom), with the peptide sequence used to produce the antibody indicated above the consensus.

An array of 384 peptides, each 18 amino acids in length with N‐terminal acetylation were synthesized and spotted onto nitrocellulose membranes (Celluspots™, http://www.intavis.com). Peptides sequences (Table S2) were designed from the concatenated sequences of 62 complete RR‐2 family CuPs from *A. pisum*, which have been annotated by Gallot *et al*. (2010). Due to the highly repetitive nature of RR‐2 CuPs, particularly the N‐ and C‐terminal domains, peptide sequences present in multiple CuPs were included only once. However, when sequences were polymorphic (in one or more amino acid positions) both versions were included in the array to cover the diversity of the RR‐2 subfamily; secretion signal peptides were not represented (see Fig. 2A). The array was printed in duplicate in a microscope slide format.

Incubations with antibodies were performed according to the manufacturer's instructions (Intavis AG, Cologne, Germany). Slides were first incubated in blocking solution prior to the addition of antibodies diluted 1∶1000 for 3 h at room temperature. Four washes in TBST buffer were carried out between primary or secondary antibodies incubations. Immunolabeling was detected by colorimetry using nitro‐blue tetrazolium and 5‐bromo‐4‐chloro‐3′‐indolyphosphate as substrate (Promega, Madison, WI, USA).

Viral proteins or viruses (5–50 μg) were incubated on blocked peptide array slides in their appropriate buffer for 24 h at 4 °C. Three rinses were then performed in the same buffer. Purified CMV particles and CMV‐CP were incubated in 50 mmol/L sodium citrate buffer (pH 7.0); whereas HP2 protein (CaMV) was incubated either in DB5 buffer or in TBST buffer used at various concentrations (either 1× , 0.5× , 0.1× ), in independent experiments. Hybridization was detected using anti‐CMV (Agdia, Elkhart, IN, USA) and anti‐P2 (Blanc *et al*., 1993a) primary antisera, and GAR‐AP secondary antiserum as described above. Hybridizations on the peptide array were quantified using standard conditions (Spotfinder v3.2.1; available online at http://www.tm4.org/spotfinder.html). The intensities of replicate spots were averaged, and considered positive when >8000 units. This threshold, defined arbitrarily, corresponds to where the intensity of the spots ranked in decreasing order, changes markedly in CMV experiments (Fig. 2A). This value also corresponds to a significant decrease in spot intensities observed visually. The corresponding peptides were finally aligned (MEGA version 4.1; Tamura *et al*., 2007) and a consensus sequence created with WebLogo 3 (www.weblogo.threeplusone.com).

## Results

### Multiple peptides across the RR‐2 chitin‐binding domain are accessible in the acrostyle

In a previous study using specific antibodies on individualized stylets (Uzest *et al*., 2010), the pepL motif was identified as being embedded in the chitin matrix at the tip of aphid maxillary stylets within the acrostyle. In order to identify motifs readily accessible at the surface of the acrostyle, including sequences directly binding the P2 protein of CaMV (Uzest *et al*., 2007), 11 additional antibodies were produced against overlapping peptides surrounding the pepL motif, with each peptide originating from different RR‐2 CuPs of *A. pisum* (Table 1, Fig. 1A).

Antibody specificity was first verified by western blot analysis through labeling of GST‐peptide fusions produced in *E. coli* (Fig. S1). The antibodies were highly specific, most of them recognizing only their related GST‐peptide fusion. However, some cross‐reactivity was observed between peptides and antibodies of Ap2‐03 and Ap2‐04, which share 12 amino acids in common, and between Ap2‐05 and Ap2‐06, which share 11 amino acids in common.

The antibodies were then used in an *in vitro* interaction assay to label dissected *A. pisum* stylets. In addition to anti‐pepL, 3 other antibodies (anti‐pepS, anti‐Ap2‐05, and anti‐Ap2‐08) were used to label the tip of maxillary stylets in the majority of observed stylets (Table 1). Peptides pepS and Ap2‐05, but not peptide Ap2‐08, were present at the surface of the acrostyle, albeit at a reduced density or accessibility, with labeling generally observed as a series of 2 or 3 spots located at the acrostyle (Fig. 1B, top row images). Furthermore, as observed previously with anti‐pepL labeling, the anti‐pepS antibody also revealed peptides accessible on the edges surrounding the acrostyle (indicated by a white arrow, Fig. 1B), indicating that the pepS peptide is distributed at least throughout the distal tip of maxillary stylets. This proximal edge was not labeled by anti‐Ap2‐05 or Ap2‐08 (Fig. 1B). As with pepL, the accessibility of these motifs was increased by removal of the surface layer of chitin by partial digestion prior to antibody interaction (Fig. 1B, bottom row images).

In contrast to the reproducible labeling seen with the other antibodies, anti‐Ap2‐03 showed only weak labeling (dots) on less than half of the maxillary stylets observed, precluding any conclusive interpretation. Therefore, this antibody was not considered as labeling the acrostyle. If present, which still awaits confirmation, this motif is likely only weakly accessible at the surface. The remaining 7 antibodies did not label any stylets under the conditions used in this study (e.g., as seen for anti‐Ap2‐09, Fig. 1B).

Taken together these results indicate that peptides pepL, pepS, Ap2‐05, and Ap2‐08, or highly similar sequences, are present within the acrostyle of *A. pisum* (Fig. 1, Table 1).

### Celluspot peptide arrays identify peptides recognized by cuticular protein antibodies

The presence of cuticular protein from the RR‐2 subfamily at the tip and acrostyle of maxillary stylets has been confirmed, both here and previously (Uzest *et al*., 2010). Therefore, an array of overlapping peptides, each 18 amino acids in length, was designed. This array of 384 peptides together represents the sequence diversity contained within this protein subfamily in *A. pisum*. The array was established in order to identify peptide motifs of RR‐2 proteins involved in direct binding with anticuticular protein antibodies or viral proteins (see overview of probe design and testing, Fig. 2A).

**Figure 3:**
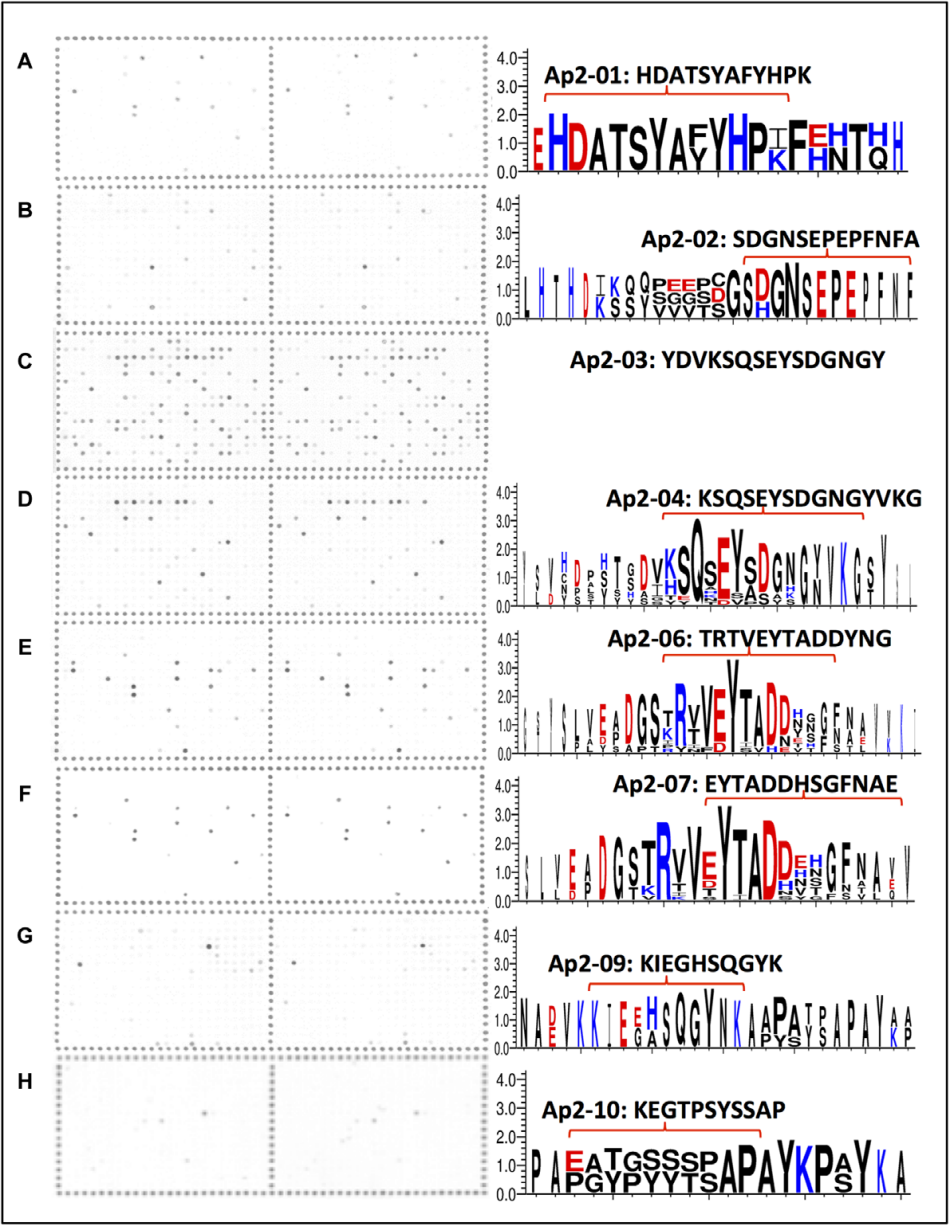
Identification of peptides recognized by cuticular protein antibodies using peptide array hybridizations. Scanned peptide arrays (left) and derived peptide consensus (right) of the hybridization for the 8 antibodies against peptides from the RR‐2 subfamily (with the peptide sequence noted above the consensus) that showed no labeling on dissected *A. pisum* stylets (results for the other 4 antibodies are presented in Fig. 2). Red brackets indicate the overlap between the peptide sequence and the consensus sequence of the hybridized peptides. Due to the high number of labeled peptides (106 peptides, Table 2) no consensus was included for Ap2‐03.

The 12 antibodies produced were hybridized to the Celluspot peptide arrays. For all antibodies, the pattern of positive spots was highly consistent between duplicate arrays, indicating specific and highly reproducible hybridization pattern. Apart from anti‐Ap2‐03, which showed low specificity hybridizing to 106 spots from which no consensus sequence could be established (Table 2, Fig. 3C), all antibodies bound strongly to a small number of spots (Table 2). The antibodies labeling the acrostyle anti‐pepS, anti‐pepL, anti‐Ap2‐05, and anti‐Ap2‐08 were highly specific and revealed 2–18 strongly labeled spots (intensity ≥12 000), and 4–19 spots considered positive (intensity ≥8000) in our analysis. The consensus sequence of these hybridizing peptides was very similar to that of the peptides used for antibody production (Fig. 2B). Furthermore, our results suggest that these antibodies recognize not only the peptide used for their production, but also other closely homologous sequences. Importantly, this shows that, even though 7 antibodies (anti‐Ap2‐01, anti‐Ap2‐02, anti‐Ap2‐04, anti‐Ap2‐06, anti‐Ap2‐07, anti‐Ap2‐09, and anti‐Ap2‐010) did not label aphid stylets in our experiments, they all bound to several peptides on the array (from 3 to 15 in total). Consensus sequences were also deduced (Fig. 3), which again closely matched the peptide used for their production. Therefore, the absence of labeling on the stylets was not due to a lack of immunogenicity of the antibodies but likely to the absence or lack of accessibility of the peptide sequence in the stylet.

**Table 2:** Specificity of labeling of @CuPs and peptides exhibiting significant binding

	Strong†	Medium‡	Weak§	Unlabeled¶
Viral protein/viruses				
CMV virions	71	41	48	224
CMV CP	0	29	63	292
Buffer	0	0	1	383
Cuticular peptides				
Ap2‐01	2	2	4	376
pepS	10	4	19	351
Ap2‐02	2	1	11	370
Ap2‐03	33	36	37	278
Ap2‐04	11	1	7	365
pepL	2	2	2	378
Ap2‐05	18	1	12	353
Ap2‐06	14	1	6	363
Ap2‐07	6	6	2	372
Ap2‐08	2	0	8	374
Ap2‐08	2	2	2	378
Ap2‐09	3	0	3	378
Buffer	0	0	0	384

^†^Binding of >12 000 units observed on average.

^‡^Binding of between 8000 and 12 000 units observed on average.

^§^Binding of between 3000 and 8000 units observed on average.

^¶^Binding of <3000 units observed on average.

### Celluspot peptide arrays to identify peptides from RR‐2 cuticular protein interacting with noncirculative viruses

The Celluspot peptide arrays approach was successfully validated with the antibodies, as described above. Subsequently, a series of assays using 2 noncirculative viruses were conducted on the arrays to investigate their putative interaction with peptides from the RR‐2 protein subfamily.

**Figure 4:**
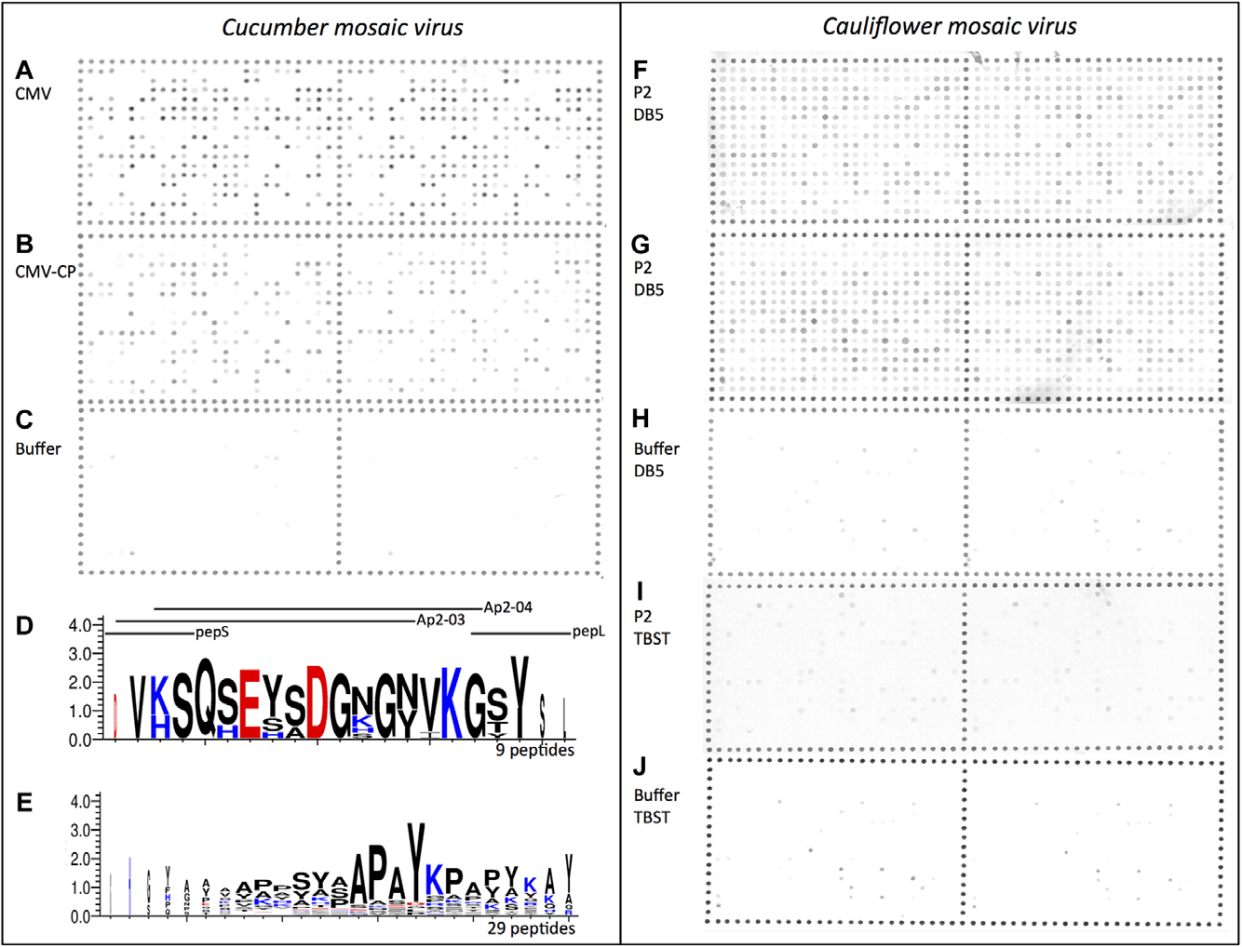
Identification of peptides from the RR‐2 protein subfamily interacting with *Cucumber mosaic virus* and the helper component protein P2 of *Cauliflower mosaic virus*. Scanned arrays hybridized with (A) *Cucumber mosaic virus* particles (CMV), (B) its coat protein (CMV‐CP), or (C) mock (buffer); (F), (G), (I) HP2 protein from *Cauliflower mosaic virus*, or (H), (J) mock (DB5 or TBST buffer, respectively). Weblogo consensus sequences obtained from the alignment of 9 (D) and 29 (E) peptides hybridizing with CMV virions. The peptide sequences used in this study that resemble or overlap with the consensus sequences are indicated above the Weblogo sequences.

First, Celluspot peptide arrays were incubated with the viral protein P2 from CaMV, used here as a His–tagged purified fusion (HP2). Strong labeling on the array was never observed with HP2 hybridization, despite evaluating several conditions for the *in vitro* interaction assay. Instead, the slides always showed nonspecific hybridizations appearing as a gradual increase in intensity across the 384 spots. In addition, the pattern of hybridization under different conditions was not reproducible (Figs. 4F–G). Therefore, no conclusion regarding the interaction of HP2 with the peptides could be reached.

Second, Celluspot peptide arrays were incubated with *Cucumber mosaic virus* (CMV). As CMV is transmitted by aphids in the form of virus particles, the latter were used in this assay. CMV coat protein (CMV‐CP) was also tested to check whether the interaction between virus particles and aphid stylets requires mature virion conformation, or simply coat protein monomers or oligomers. In the latter case, interaction on the arrays using CMV‐CP or derived peptides could help define the viral amino acids directly involved in cuticular peptide(s) binding. When purified CMV virions and CMV‐CP were incubated on the arrays, a highly specific and repeatable pattern of hybridization was observed for both viral compounds. However, overall hybridization intensities and therefore numbers of positive spots were greater for intact virions than for CP alone (112 peptides vs. 29 peptides, see Figs. 4A, B and Table 2). Mock (citrate buffer) incubated slides showed hybridization at a single peptide only (peptide 341, Fig. 4C, Table 2), which was excluded from further analysis. From hybridizations with intact virions, 2 peptide consensuses were developed from 9 and 29 peptides of similar sequences (Figs. 4D–E). The first corresponds to a sequence partially overlapping the pepS and pepL peptide sequences and strongly resembling peptides Ap2‐03 and Ap2‐04 (Fig. 4D). The second peptide is a repeat‐rich sequence containing proline, alanine, tyrosine, and serine typically present in the N‐ and C‐terminii of RR‐2 proteins (Fig. 4E).

## Discussion

Here we show that multiple regions across the RR‐2 domain of cuticular proteins are accessible in the acrostyle. Indeed, our series of antibodies covered the entire RR‐2 domain, and 4 peptides from the N‐ and C‐terminal end of the chitin‐binding domain were clearly labeled. These accessible peptides were not contiguous, but instead were separated by a few amino acids that we could not detect on the surface of the stylets, even following chitinase treatment (Fig. 1B) and despite using multiple antibodies. These amino acid sequences were undetected presumably because of a lack of accessibility to the antibody; undetected sequences might possibly be involved in interactions with chitin fibers or other cuticular proteins in the stylet cuticle and therefore be unavailable for binding by antibodies. Indeed, the whole RR‐2 domain has been reported to be involved in interaction and stabilization of chitin fibers (Iconomidou *et al*., 1999; Rebers & Willis, 2001; Hamodrakas *et al*., 2002; Vincent & Wegst, 2004). Alternatively, RR‐2 protein folding could mask epitopes recognized by the antibodies, or the undetected sequences could be buried deeper within the chitin matrix than the partial chitinase digestion could reach. The absence of labeling of stylets with the anti‐Ap2‐06 antibody, while strong labeling was seen with the very similar anti‐Ap2‐05 antibody, was unexpected. Indeed, Ap2‐05 and Ap2‐06 share more than 84% identity (11 identical amino acids out of 13), and anti Ap2‐06 antibody recognized both Ap2‐05 and Ap2‐06 peptides when present on artificial membranes. This result might indicate that the 2 residues, a histidine (H) and a serine (S), located at the C‐terminus of the peptide sequence, are accessible in the acrostyle after partial chitinase digestion. It might also suggest that the H ad S residues are important for a recognition by anti‐Ap2‐05 antibody in the acrostyle context.

The series of antibodies developed here should be able to detect most, if not all, of the 78 RR‐2 CuPs described by Gallot *et al*. (2010). However, the presence of CuPs from the RR‐2 subfamily could be detected only within a small region at the tip of aphid maxillary stylets, in the acrostyle, and on 1 side edge at the tip of the stylet (clearly visible in Fig. 1B), as previously shown for the anti‐pepL antibody (Uzest *et al*., 2010). Whether these observations reflect a lack of accessibility of protein sequences at the surface of the cuticle, or whether the remainder of the stylet cuticle contains cuticular proteins from other families, remains to be determined.

The accessibility of all peptides detected in the acrostyle was increased when stylets were treated with chitinase (Fig. 1B), indicating that the RR‐2 domain is anchored mainly within the cuticle, and is only poorly accessible at the surface. Strong labeling of the acrostyle has never been observed without prior enzymatic treatment. This suggests that if a cuticular protein from the RR‐2 subfamily is involved in CaMV transmission, then its virus‐binding domain might not be a peptide sequence located within the chitin‐binding region, but is more likely located in the N‐ or C‐terminal part of the protein. Indeed, the P2 viral protein binds strongly all over the acrostyle (Uzest *et al*., 2007) without chitinase treatment.

The identity of specific RR‐2 proteins present at the acrostyle could not be determined due to the inherent limitations of the antibody method. Cross‐reactivity with nearly identical sequences was observed in our experiments (e.g., anti‐Ap2‐05 reacted with peptide Ap2‐06 in our study, Fig. S1; numerous spots obtained on the arrays, Fig. 3). For this reason, the exact sequence recognized *in situ* (in the stylets) by a specific antibody could not be assessed accurately. Thus, although the presence of ACYPI004893 protein alone is sufficient to explain the labeling observed in the acrostyle (this protein contains perfect matches to the peptide sequences recognized by the 4 antibodies for which we observed labeling: anti‐pepL, anti‐pepS, anti‐pepAp2‐05, and anti‐pepAp2‐08), the presence of one or more other proteins cannot be excluded, and needs additional testing. The highly repetitive nature of the RR‐2 subfamily, particularly the N‐ and C‐ terminals with their repeats of proline, alanine, serine and tyrosine, make production of specific antibodies for each RR‐2 protein unfeasible; therefore other approaches will be required to identify receptor candidates.

The propensity of RR‐2 subfamily peptides to interact with noncirculative viruses was also assessed using a nonrepeating array of 18 amino acid peptides. Unfortunately, results obtained with HP2 were inconsistent, and therefore no conclusion could be made regarding peptides that may be involved in CaMV binding. The P2 protein is known to require high specific salt conditions for solubility (e.g., DB5 buffer containing 500 mmol/L lithium chloride), and often precipitates upon concentration (Blanc *et al*., 1993b; Hébrard *et al*., 2001). Conditions used in this study might have been inappropriate for interaction assays when hybridizations were done in high salt concentration to favor P2 solubility (DB5 buffer, Figs. 4G, H); or detrimental to the biological function of P2 when interactions were assessed in classical TBST hybridization buffer (Fig. 4I). In contrast, results obtained with CMV were reliable both when using mature and disrupted virions. However, CMV‐CP bound fewer peptides than intact virus particles, suggesting that capsid conformation of intact virions favored binding of CP to cuticular peptides on the arrays. Two consensus motifs binding to CMV could be deduced from the results obtained with CMV particles (Fig. 4). One of these 2 motifs indicates that peptides interacting with CMV are present within the RR‐2 domain, in close proximity to the pepS peptide, and hence could be accessible at the surface of the acrostyle. The second consensus sequence, a highly repeated sequence of proline, alanine, serine and tyrosine residues, is present at the N‐ and C‐terminal domains of the majority of the RR‐2 proteins. While its presence at the surface was not demonstrated conclusively, it is potentially at or near the surface of the acrostyle because peptide Ap2‐08 (which was revealed in the acrostyle by immunolabeling with anti Ap2‐08) is adjacent to it, and is located at the C‐terminal end of the RR‐2 domain. These conclusions are based on *in vitro* interaction assays and might not be biologically relevant. Therefore, additional biochemical and functional validation will be required to make any conclusions regarding the role of these motifs in the transmission of CMV and other noncirculative viruses.

Through the 2 novel approaches developed here for investigating insect–virus interactions, we further characterized the proteomic composition of the acrostyle and the domains of RR‐2 proteins that were accessible and potentially available to interact with components flowing into the stylets’ common canal. Remarkably, consensus sequences deduced from the hybridizations of the peptide array with antibodies specific for RR‐2 cuticular peptides match closely the initial peptide sequences used for the production of these antibodies. Our peptide array approach is thus validated as a powerful tool to search for cuticular protein sequences that interact with any protein of interest. Beyond the scope of this study, this approach may have broader applications in seeking putative interacting partners of R&R cuticular proteins, thus contributing to a better understanding of the physiological and biochemical properties of cuticular proteins, other than their role as structural constituent of the arthropods exoskeleton.

## Disclosure

The authors declare that they have no conflicts of interest.

## Supporting information

**Fig. S1** Antibodies reactivity against GST‐peptide fusions. (A) Schematic representation of GST‐peptide fusions with 3 of the peptides used to produce antibodies, and expressed in *Escherichia coli* (*E. coli*). (B) Crude extracts of the 11 GST‐peptide fusions produced in *E. coli* were loaded on a SDS‐PAGE. Crude extracts of empty plasmid (GST), or empty bacteria (*E. coli*) were used as negative controls. After electrophoresis, gels were either stained with Coomassie blue, or transferred onto a nitrocellulose membrane for specific detection by western blot analysis using the corresponding 11 antipeptide antibodies. All antibodies showed high specificity labeling of the corresponding GST‐peptide fusion (expected fusions size being 27–28 kDa, black arrow, GST alone being 26 kDa, grey arrow), or their close related ones. In some cases, additional bands of lower size were also detected, probably reflecting partial degradation of the GST‐peptide fusions. Anti‐Ap2‐07 was produced recently. The GST‐pepAp2‐07 plasmid was not yet available; therefore, the analysis has not been conducted for this fusion. However, the specificity of the anti‐Ap2‐07 antibody was assessed using the peptide array approach (see Fig. 3). The position of molecular weight markers is indicated on the left of western blots.
**Table S1**. Oligonucleotides used for pGEX‐3X‐peptide fusions.
**Table S2**. Sequences of peptides spotted on the peptide array and their hybridization to RR‐2 subfamily antibodies, or CMV proteins.

## References

[ins12469-bib-0001] Andersen, S.O. , Hojrup, P. and Roepstorff, P. (1995) Insect cuticular proteins. Insect Biochem and Molecular Biology, 25, 153–176. 10.1016/0965-1748(94)00052-j 7711748

[ins12469-bib-0002] Blanc, S. , Cerutti, M. , Usmany, M. , Vlak, J.M. and Hull, R. (1993a) Biological activity of cauliflower mosaic virus aphid transmission factor expressed in a heterologous system. Virology, 192, 643–650. 8421904 10.1006/viro.1993.1080

[ins12469-bib-0003] Blanc, S. , Drucker, M. and Uzest, M. (2014) Localizing viruses in their insect vectors. Annual Review of Phytopathology, 52, 403–425. 10.1146/annurev-phyto-102313-045920 24996011

[ins12469-bib-0004] Blanc, S. and Michalakis, Y. (2016) Manipulation of hosts vectors by plant viruses and impact on the environment. Current Opinion in Insect Science, 16, 36–43. 27720048 10.1016/j.cois.2016.05.007

[ins12469-bib-0005] Blanc, S. , Schmidt, I. , Kuhl, G. , Esperandieu, P. , Lebeurier, G. , Hull, R. *et al* (1993b) Paracrystalline structure of cauliflower mosaic virus aphid transmission factor produced both in plants and in a heterologous system and relationship with a solubilized active form. Virology, 197, 283–292. 8212564 10.1006/viro.1993.1589

[ins12469-bib-0006] Brault, V. , Uzest, M. , Monsion, B. , Jacquot, E. and Blanc, S. (2010) Aphids as transport devices for plant viruses. Comptes Rendus Biologies, 333, 524–538. 20541164 10.1016/j.crvi.2010.04.001

[ins12469-bib-0007] Chen, B. and Francki, R.I.B. (1990) Cucumovirus transmission by the aphid *Myzus persicae* is determined solely by the viral coat protein. Journal of General Virology, 71, 939–944.

[ins12469-bib-0008] Cornman, R.S. , Togawa, T. , Dunn, W.A. , He, N. , Emmons, A.C. and Willis, J.H. (2008) Annotation and analysis of a large cuticular portein family with the R&R consensus in *Anopheles gambiae* . BMC Genomics, 18, 9–22. 10.1186/1471-2164-9-22 PMC2259329 18205929

[ins12469-bib-0009] Dedryver, C.A. , Le Ralec, A. and Fabre, F. (2010) The conflicting relationships between aphids and men: a review of aphid damage and control strategies. Comptes Rendus Biologies, 333, 539–553. 20541165 10.1016/j.crvi.2010.03.009

[ins12469-bib-0010] Dixon, A.F.G. (1998) Aphid Ecology: An Optimization Approach. 2nd ed. Chapman and Hall, London.

[ins12469-bib-0011] Gallot, A. , Rispe, C. , Leterme, N. , Gauthier, J.P. , Jaubert‐Paussamai, S. and Tagu, D. (2010) Cuticular proteins and seasonal photoperiodism in aphids. Insect Biochemistry and Molecular Biology, 40, 235–240. 20018241 10.1016/j.ibmb.2009.12.001

[ins12469-bib-0012] Gera, A. , Loebenstein, G. and Raccah, B. (1979) Protein coats of two strains of *Cucumber mosaic virus* affect transmission of *Aphis gossypii* . Phytopathology, 69, 369–399.

[ins12469-bib-0013] Hamodrakas, S.J. , Willis, J.H. and Iconomidou, V.A. (2002) A structural model of the chitin‐binding domain of cuticle proteins. Insect Biochemistry and Molecular Biology, 32, 1577–1583. 12530225 10.1016/s0965-1748(02)00079-6

[ins12469-bib-0014] Hébrard, E. , Drucker, M. , Leclerc, D. , Hohn, T. , Uzest, M. , Froissart, R. *et al* (2001) Biochemical characterization of the helper component of *Cauliflower mosaic virus* . Journal of Virology, 75, 8538–8546. 11507199 10.1128/JVI.75.18.8538-8546.2001 PMC115099

[ins12469-bib-0015] Hogenhout, S.A. , Ammar el, D. , Whitfield, A.E. and Redinbaugh, M.G. (2008) Insect vector interactions with persistently transmitted viruses. Annual Review of Phytopathology, 46, 327–359. 10.1146/annurev.phyto.022508.092135 18680428

[ins12469-bib-0016] Iconomidou, V.A. , Willis, J.H. and Hamodrakas, S.J. (1999) Is beta‐pleated sheet the molecular conformation which dictates formation of helicoidal cuticle? Insect Biochemistry and Molecular Biology, 29, 285–292. 10319442 10.1016/s0965-1748(99)00005-3

[ins12469-bib-0017] Jaouannet, M. , Rodriguez, P.A. , Thorpe, P. , Lenoir, C.J.G , MacLeod, R. , Escudero‐Martinez, C. , *et al* (2014) Plant immunity in plant‐aphid interactions. Frontiers in Plant Science, 5, 663, 1–10. 10.3389/fpls.2014.00663 PMC4249712 25520727

[ins12469-bib-0018] Kaloshian, I. and Walling, L.L. (2016) Hemipteran and dipteran pests: effectors and plant host immune regulators. Journal of Integrative Plant Biology, 58, 350–361. 26467026 10.1111/jipb.12438

[ins12469-bib-0019] Kaper, J.M. (1969) Reversible dissociation of *Cucumber mosaic virus* (strain S). Virology, 37, 134–139. 5762199 10.1016/0042-6822(69)90315-8

[ins12469-bib-0020] Laemmli, U.K. (1970) Cleavage of structural proteins during the assembly of the head of bacteriophage T4. Nature (London), 277, 680–684. 10.1038/227680a0 5432063

[ins12469-bib-0021] Lot, H. , Marrou, J. , Quiot, J.B. and Esvan, C. (1972) Contribution à l'étude du virus de la mosaïque du concombre (CMV). I. Méthode de purification rapide du virus. Annales De Phytopathologie, 4, 25–38.

[ins12469-bib-0022] Lu, X. , Thompson, J.R. and Perry, K.L. (2012) Encapsidation of DNA, a protein and a fluorophore into virus‐like particles by the capsid protein of *Cucumber mosaic virus* . Journal of General Virology, 93, 1120–1126. 22278829 10.1099/vir.0.040170-0

[ins12469-bib-0023] Martin, B. , Collar, J.L. , Tjallingii, W.F. and Fereres, A. (1997) Intracellular ingestion and salivation by aphids may cause the acquisition and inoculation of non‐persistently transmitted plant viruses. Journal of General Virology, 78, 2701–2705. 9349493 10.1099/0022-1317-78-10-2701

[ins12469-bib-0024] Mugford, S.T. , Barclay, E. , Drurey, C. , Findlay, K.C. and Hogenhout, S.A. (2016) An immuno‐suppressive aphid saliva protein is delivered into the cytosol of plant mesophyll cells during feeding. Molecular Plant–Microbe Interactions, 29, 854–861 27831211 10.1094/MPMI-08-16-0168-R

[ins12469-bib-0025] Nicholson, S.J. , Hartson, S.D. and Puterka, G.J. (2012) Proteomic analysis of secreted saliva from Russian wheat aphid (*Diuraphis noxia* Kurd.) biotypes that differ in virulence to wheat. Journal of Proteomics, 75, 2252–2268. 22348819 10.1016/j.jprot.2012.01.031

[ins12469-bib-0026] Powell, G. (2005) Intracellular salivation is the aphid activity associated with inoculation of non‐persistently transmitted viruses. Journal of General Virology, 86, 469–472. 15659767 10.1099/vir.0.80632-0

[ins12469-bib-0027] Rebers, J.E. and Riddiford, L.M. (1988) Structure and expression of a *Manduca sexta* larval cuticle gene homologous to Drosophila cuticle genes. Journal of Molecular Biology, 203, 411–423. 2462055 10.1016/0022-2836(88)90009-5

[ins12469-bib-0028] Rebers, J.E. and Willis, J.H. (2001) A conserved domain in arthropod cuticular proteins binds chitin. Insect Biochemistry and Molecular Biology, 31, 1083–1093. 11520687 10.1016/s0965-1748(01)00056-x

[ins12469-bib-0029] Tagu, D. , Klingler, J.P. , Moya, A. and Simon, J.‐C. (2008) Early progress in aphid genomics and consequences for plant‐aphid interactions studies. Molecular Plant–Microbe Interactions, 21, 701–708. 18624634 10.1094/MPMI-21-6-0701

[ins12469-bib-0030] Tamura, K. , Dudley, J. , Nei, M. and Kumar, S. (2007) MEGA4: Molecular Evolutionary Genetics Analysis (MEGA) software version 4.0. Molecular Biology and Evolution, 24, 1596–1599. 17488738 10.1093/molbev/msm092

[ins12469-bib-0031] Uzest, M. and Blanc, S. (2016) Molecular mechanisms involved in noncirculative virus–vector interactions Vector‐Mediated Transmissions of Plant Pathogen (ed. Brown J.K.). San Diego, American Phytopathological Society Press, Saint Paul, USA.

[ins12469-bib-0032] Uzest, M. , Gargani, D. , Dombrovsky, A. , Cazevieille, C. , Cot, D. and Blanc, S. (2010) The “acrostyle”: a newly described anatomical structure in aphid stylets. Arthropod Structure & Development, 39, 221–229. 20170746 10.1016/j.asd.2010.02.005

[ins12469-bib-0033] Uzest, M. , Gargani, D. , Drucker, M. , Hébrard, E. , Garzo, E. , Candresse, T. , *et al* (2007) A protein key to plant virus transmission at the tip of the insect vector stylet. Proceedings of the National Academy of Sciences of the United States of America, 104, 17959–17964. 17962414 10.1073/pnas.0706608104 PMC2084279

[ins12469-bib-0034] Vincent, J.F. and Wegst, U.G. (2004) Design and mechanical properties of insect cuticle. Arthropod Structure & Development, 33, 187–199. 18089034 10.1016/j.asd.2004.05.006

[ins12469-bib-0035] Willis, J.H. (2010) Structural cuticular proteins from arthropods: annotation, nomenclature, and sequence characteristics in the genomics era. Insect Biochemistry and Molecular Biology, 40, 189–204. 20171281 10.1016/j.ibmb.2010.02.001 PMC2872936

