# Supplementary figures and images for: Proteomic composition of the acrostyle: Novel approaches to identify cuticular proteins involved in virus–insect interactions
Source: Insect Sci. 2017 Jun 22;24(6):990–1002. doi: 10.1111/1744-7917.12469 (PMC5724696; doi:10.1111/1744-7917.12469)

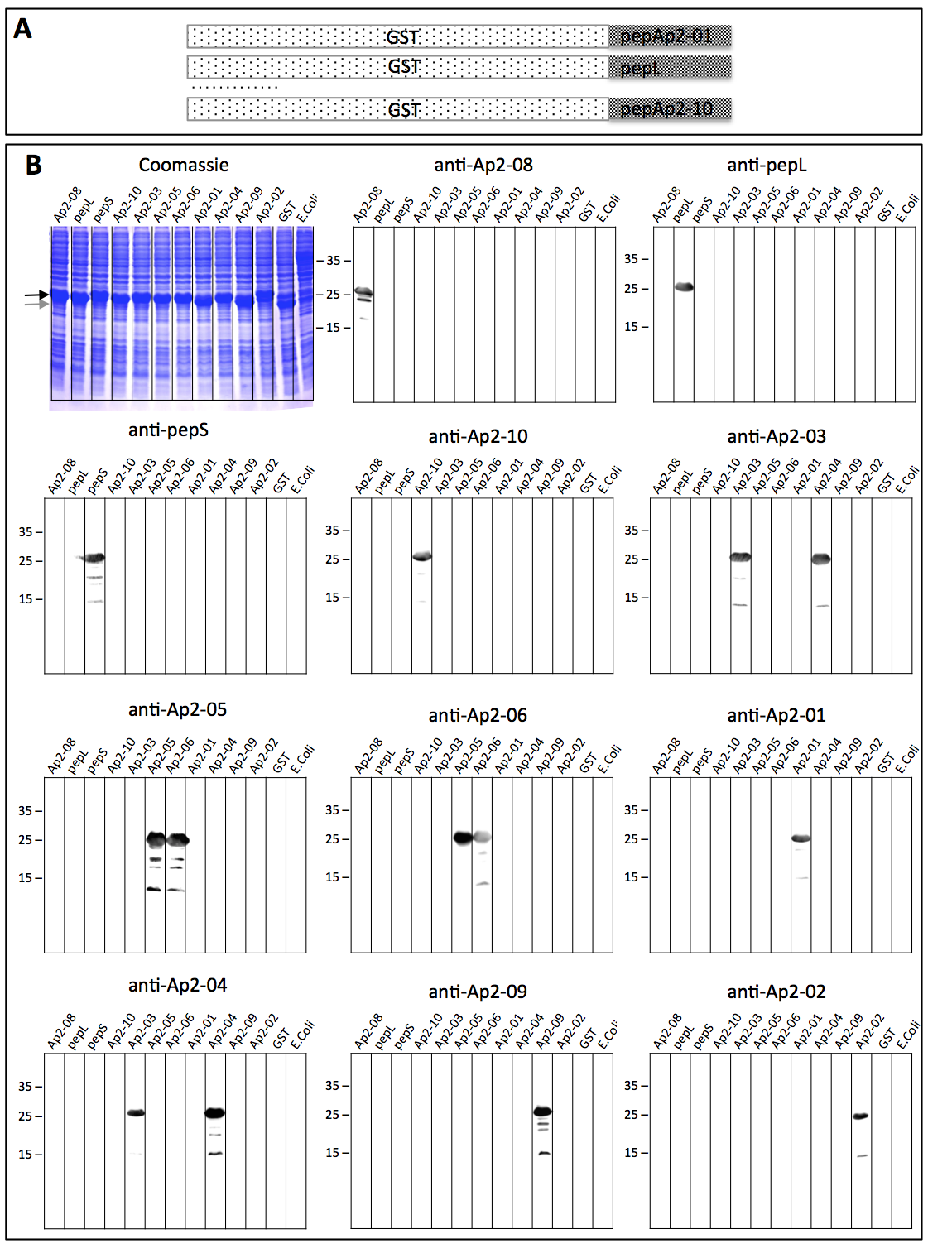

Supplement: Supplementary file 1 [file INS-24-990-s001.tiff]
