# Supplementary material for: Proteomic composition of the acrostyle: Novel approaches to identify cuticular proteins involved in virus–insect interactions
Source: Insect Sci. 2017 Jun 22;24(6):990–1002. doi: 10.1111/1744-7917.12469 (PMC5724696; doi:10.1111/1744-7917.12469)
Supplement: Supplementary file 2 — Fig. S1 Antibodies reactivity against GST‐peptide fusions. (A) Schematic representation of GST‐peptide fusions with 3 of the peptides used to produce antibodies, and expressed in Escherichia coli (E. coli). (B) Crude extracts of the 11 GST‐peptide fusions produced in E. coli were loaded on a SDS‐PAGE. Crude extracts of empty plasmid (GST), or empty bacteria (E. coli) were used as negative controls. After electrophoresis, gels were either stained with Coomassie blue, or transferred onto a nitrocellulose membrane for specific detection by western blot analysis using the corresponding 11 antipeptide antibodies. All antibodies showed high specificity labeling of the corresponding GST‐peptide fusion (expected fusions size being 27–28 kDa, black arrow, GST alone being 26 kDa, grey arrow), or their close related ones. In some cases, additional bands of lower size were also detected, probably reflecting partial degradation of the GST‐peptide fusions. Anti‐Ap2‐07 was produced recently. The GST‐pepAp2‐07 plasmid was not yet available; therefore, the analysis has not been conducted for this fusion. However, the specificity of the anti‐Ap2‐07 antibody was assessed using the peptide array approach (see Fig. 3). The position of molecular weight markers is indicated on the left of western blots. Table S1. Oligonucleotides used for pGEX‐3X‐peptide fusions. Table S2. Sequences of peptides spotted on the peptide array and their hybridization to RR‐2 subfamily antibodies, or CMV proteins. [file INS-24-990-s002.docx]

**Fig. S1:** Antibodies reactivity against GST-peptide fusions**.** A). Schematic representation of GST-peptide fusions with 3 of the peptides used to produce antibodies, and expressed in *Escherichia coli* (*E.coli*). B). Crude extracts of the 11 GST-peptide fusions produced in *E.coli* were loaded on a SDS-PAGE. Crude extracts of empty plasmid (GST), or empty bacteria (*E.*coli) were used as negative controls. After electrophoresis, gels were either stained with Coomassie blue, or transferred onto a nitrocellulose membrane for specific detection by western blot analysis using the corresponding 11 anti-peptide antibodies. All antibodies showed high specificity labeling of the corresponding GST-peptide fusion (expected fusions size being 27 to 28 kDa, black arrow, GST alone being 26 kDa, grey arrow), or their close related ones. In some cases, additional bands of lower size were also detected, probably reflecting partial degradation of the GST-peptide fusions. Anti-Ap2-07 was produced recently. The GST-pepAp2-07 plasmid was not yet available, therefore the analysis has not been conducted for this fusion. However, the specificity of the anti-Ap2-07 antibody was assessed using the peptide array approach (see Fig. 3). The position of molecular weight markers is indicated on the left of western blots.

**Table S1.** Oligonucleotides used for pGEX-3X-peptide fusions

Oligonucleotide DNA sequence (5’ to 3’)

pGEX-Ap2-01 for GATCCccCACGACGCTACATCCTACGCGTTTTACCATCCGAAAG

pGEX-Ap2-01 rev AATTCTTTCGGATGGTAAAACGCGTAGGATGTAGCGTCGTGggG

pGEX-pepS for GATCCccTTCGAATACAGCGTAAACGACCCACACACCTACGATGTCAAGAGCG

pGEX-pepS rev AATTCGCTCTTGACATCGTAGGTGTGTGGGTCGTTTACGCTGTATTCGAAggG

pGEX-Ap2-02 for GATCCccTCAGATGGAAATTCAGAACCCGAGCCGTTCAACTTTGCTG

pGEX-Ap2-02 rev AATTCAGCAAAGTTGAACGGCTCGGGTTCTGAATTTCCATCTGAggG

pGEX-Ap2-03 for GATCCccTACGATGTCAAGAGCCAATCTGAATACAGCGACGGAAACGGTTACG

pGEX-Ap2-03 rev AATTCGTAACCGTTTCCGTCGCTGTATTCAGATTGGCTCTTGACATCGTAggG

pGEX-Ap2-04 for GATCCccAAGAGCCAATCTGAATACAGCGACGGAAACGGTTACGTCAAGGGAG

pGEX-Ap2-04 rev AATTCTCCCTTGACGTAACCGTTTCCGTCGCTGTATTCAGATTGGCTCTTggG

pGEX-pepL for GATCCccGGATCGTACAGCCTTTTGGAAGCCGACGGTTCCACCCGTACCGTCG

pGEX-pepL rev AATTCGACGGTACGGGTGGAACCGTCGGCTTCCAAAAGGCTGTACGATCCggG

pGEX-Ap2-05 for GATCCccACCCGTACCGTAGAATACACCGCCGATGACCACAGCGGTG

pGEX-Ap2-05 rev AATTCACCGCTGTGGTCATCGGCGGTGTATTCTACGGTACGGGTggG

pGEX-Ap2-06 for GATCCccACCCGTACTGTAGAATACACCGCTGACGACTACAACGGTG

pGEX-Ap2-06 rev AATTCACCGTTGTAGTCGTCAGCGGTGTATTCTACAGTACGGGTggG

pGEX-Ap2-08 for GATCCccGTCGTCAAGAACGAAGGTGGATACAAGGCCCCATCATACTCCGCAG

pGEX-Ap2-08 rev AATTCTGCGGAGTATGATGGGGCCTTGTATCCACCTTCGTTCTTGACGACggG

pGEX-Ap2-09 for GATCCccAAAATCGAAGGACACAGCCAAGGTTACAAG

pGEX-Ap2-09 rev AATTCCTTGTAACCTTGGCTGTGTCCTTCGATTTTggG

pGEX-Ap2-10 for GATCCccAAAGAAGGCACACCGTCTTACAGCTCCGCACCG

pGEX-Ap2-10 rev AATTCCGGTGCGGAGCTGTAAGACGGTGTGCCTTCTTTggG

5’ and 3’ overhanging sequences of BamHI and EcoRI restrictions sites are underlined. Bases added to keep the open reading frame are in lower cases.

**Table S2**. Sequences of peptides spotted on the peptide array and their hybridization to RR-2 subfamily antibodies, or CMV proteins.

Name Location^†^ Sequence Anti-CuP binding^‡^ CMV binding^§^

pep1 A 1 QYSAPAYKPAYSAPAYSA virions

pep2 A 2 QYSAPAYKPAYSSPAYAA virions

pep3 A 3 QYAAPAYKPAYSAPAYSA virions

pep4 A 4 QYAAPAYPAHHAEAEHAY

pep5 A 5 QYVVPAYPAAPTAYHLSP

pep6 A 6 QYAAYAPEPKYAPAPYSF

pep7 A 7 QYPAPAYKPAYPAPAYAA

pep8 A 8 QYAAPEYPAHHAYAQAEQ

pep9 A 9 DYSAPAYPAHHAYAAEHS

pep10 A10 VYKEPSYPSYPAAPAYPA

pep11 A11 QYSAPAYKPAFSIPVYSA

pep12 A12 SFPSLNDFDEHDEHQHHS

pep13 A13 YPTSVISHNGFETNHYDD Ap2-03

pep14 A14 YPPTIVEHNSYEHHDDGH Ap2-03

pep15 A15 QYAAPAYPAQHAYAQAEH

pep16 A16 EGGYAAPSPSPATYKPAY virions

pep17 A17 NQAPAYSSHQAPAYQAHG

pep18 A18 GPVAPAYPAPSAYPAPSA

pep19 A19 GSPVYPSPAYSAAPAYHA

pep20 A20 QYPAPAYKPAYPAPAYAA

pep21 A21 QYPAPAYKPAYSAPAYSA virions

pep22 A22 VPAYPAPAAAYPAPAAAY

pep23 A23 APAYPTPAAYPAPAAAYP

pep24 A24 VYKEPTYPAAAPAYSSNQ

pep25 B 1 QYYPAAAYPSTAVVHASP

pep26 B 2 SPVYTAAPYAAYPYEAAP

pep27 B 3 YPPIEYKSYDANHYDHAP

pep28 B 4 YPPEYKSYDDGHHDHYAH

pep29 B 5 YPPEHKSYYEEDDSYDGA

pep30 B 6 YPPIEYKSYDADLHYDHA

pep31 B 7 TADDEHGFRAEVKRIEPV virions

pep32 B 8 YPPTEYKSYDASHYDDHA

pep33 B 9 SYLAHRAAAYHGAAPAAI virions

pep34 B10 EHDATSYAFYHPKFENTH Ap2-01

pep35 B11 SPFQLHDHEYHPQQGHYD

pep36 B12 GDSSHKPGSTYHFQYAVH

pep37 B13 EVGDGHGSVRGTYSLVEP

pep38 B14 YPQGYGGGGGHDDDDHVD

pep39 B15 QYGSPAGNSNPGYGGTGT

pep40 B16 EGGYKAPYSAPAPAYKAA virions

pep41 B17 HDATSYAYYHPIFHHTQH virions

pep42 B18 YAVHDPLTGDEKSQNEVG Ap2-03

pep43 B19 QYGSNFGDSIPGYGGTGI Ap2-03

pep44 B20 GYNFGYGVNDPATGDIKD Ap2-03

pep45 B21 KGSYSLLEADGSTRTVEY pepL

pep46 B22 APEPAYAPAPYNFEYSVN pepS

pep47 B23 YAPEPTYAPKPYSFEYSN

pep48 B24 APEPAYAPTPYNFEYSVN pepS

Name Location^†^ Sequence Anti-CuP binding^‡^ CMV binding^§^

pep49 C 1 EHSAPTPYNFEYSVSDPH

pep50 C 2 DDGHYAHAPTPYHFEYAV

pep51 C 3 PVYSAPRAYAPEPAYAPA

pep52 C 4 PEPAYPPKPYNFEYSVND pepS

pep53 C 5 APEPAYAPAPYSFEYSVN pepS

pep54 C 6 APKPYHFEYGVKDLHTHD

pep55 C 7 DVHSQSEYSDGNGYVKGS Ap2-03,Ap2-04 virions

pep56 C 8 DVHSQSEHSDGYGNVKGT Ap2-03 virions

pep57 C 9 VKSQSEYADGNGYVKGSY Ap2-03,Ap2-04 coat protein, virions

pep58 C10 VKSQSEHSDGHGNVKGSY Ap2-03 virions

pep59 C11 VHSQSEYSDGKGYVKGTY Ap2-03,Ap2-04 coat protein, virions

pep60 C12 VHSQSESSDGNGNVKGTY Ap2-03,Ap2-04 virions

pep61 C13 VHSQSESSDGKGNVKGTY virions

pep62 C14 VKSQHEYSDGNGYVKGSY Ap2-03,Ap2-04 virions

pep63 C15 YSVNDPSTYDVKSQSEYA pepS,Ap2-03,Ap2-04

pep64 C16 FEYSVHDDATYDIKSQSE pepS,Ap2-03

pep65 C17 LHTHDIKSQHEVSDGHGN Ap2-03

pep66 C18 LHTHDIKSQSEVSDGHGN Ap2-02,Ap2-03,Ap2-04

pep67 C19 LHTHDIKSQHESSDGHGN Ap2-03

pep68 C20 PAYKPAYAAPAYPAPAAY

pep69 C21 SAPAYSAPAYKPAYKPAY virions

pep70 C22 PAHSYAAPAYKPAPYKAY coat protein, virions

pep71 C23 PAPAYSAPAYKPAPYKAY coat protein, virions

pep72 C24 APAYSAPAYPAPAYKPAY virions

pep73 D 1 SAPAPAYKPAPAPYKPAY virions

pep74 D 2 SAPAYSAPAYSAPAYSAP

pep75 D 3 KAPAYAAPAYKPAYAAPA

pep76 D 4 SAPAYAAPAYSAPAYAAP

pep77 D 5 GYKAPAYAAPAYAAPAYS

pep78 D 6 YSSPAYAAPAYAAPKAYA virions

pep79 D 7 FNAVVKNSAPSAAYKPAY Ap2-03 virions

pep80 D 8 DGSTRVVDYTADSYGFNA Ap2-03, Ap2-07

pep81 D 9 YGFNAEVKKIEGHGYSAS

pep82 D10 GYSASAPAYKSAPAYKPA virions

pep83 D11 YAAPAYKPAYKPAYKPAY coat protein, virions

pep84 D12 AYAAPAHSYAAPAYSAPA

pep85 D13 KSQHESSDGSGNVKGYYS coat protein, virions

pep86 D14 GYKPAYSAPSYSAYKPAY virions

pep87 D15 NAVVKNEGGYKAPSYSAP Ap2-08

pep88 D16 GFNAIVKNSAPAAGYKPA virions

pep89 D17 PAYSAPAYAAPAYPAPAY Ap2-09

pep90 D18 NAEVKKIEGHSQGYNAPA Ap2-09

pep91 D19 NAEVKKEGGYPAPAYSAP

pep92 D20 VKGTYSLVEADGSIRTVE pepL

pep93 D21 YKAVYPAPAYKPAPYKAY coat protein, virions

pep94 D22 YSAPAHSAPAYSAPAHSA

pep95 D23 PAYKPAYSAPTYSAPKAY coat protein, virions

pep96 D24 PAYHAPAAYSAPAYHAPA

pep97 E 1 HAPADYSAPAPYSFEYSV

pep98 E 2 VVEYTADNYGFNAEVKKI Ap2-05,Ap2-06 virions

pep99 E 3 QSEYADANGYVKGSYSLL Ap2-03,Ap2-05,Ap2-06 virions

Name Location^†^ Sequence Anti-CuP binding^‡^ CMV binding^§^

pep100 E 4 YHLSPSYHVAPAYQVAPV Ap2-03,Ap2-05,Ap2-06

pep101 E 5 QVAPVYVAAPAYQVTPAK virions

pep102 E 6 VTPAKAYKPVEPTNAPTP

pep103 E 7 NAPTPYSFVYSVNNPTTY pepS

pep104 E 8 DVKSQAESSDGNGNVKGF virions

pep105 E 9 GYASAAYKPAAAAPYKSY virions

pep106 E10 DDYSGFNAIVKKEGGYAS Ap2-05 virions

pep107 E11 VKSQSEYADGNGNIKGSY

pep108 E12 APYSFEYSVNDPTTYDVK

pep109 E13 VVKKIEGGYKAPYSAPAY Ap2-03,Ap2-05,Ap2-06

pep110 E14 TRVVEYTADDNTGFNAVV Ap2-03,Ap2-05,Ap2-06, Ap2-07

pep111 E15 QSYAPAPAYSPAAYTSSK virions

pep112 E16 AAYPAPAYSAPKSYAPEP

pep113 E17 YAVNDPHTHDIKSHHELN pepS

pep114 E18 HHELNDGHGNVKGSYSLL virions

pep115 E19 LATSYHEPIHHSYKPYQY virions

pep116 E20 TRVVTYTADHEHGFNAEV Ap2-07

pep117 E21 EVKKIEAPAHHHYEAPLA

pep118 E22 YNGFNAEVKNSAPAYKPA virions

pep119 E23 APAHSASAYSAPAYKPAY virions

pep120 E24 YSAPAYPAAPAYPAAPAY

pep121 F 1 SQSEYSDGNGNVKGSYSL Ap2-03,Ap2-04 virions

pep122 F 2 LEADGSTRVVEYTADDHS Ap2-01,Ap2-05,Ap2-06,Ap2-07

pep123 F 3 HSQGYKAPYSTPAPAYKP Ap2-09 virions

pep124 F 4 SAPAHSAQAYSAPAHSAP

pep125 F 5 YKAPYSAPSPAYRPTPYK virions

pep126 F 6 PAYSTPAHAAPAYPAPAH

pep127 F 7 PAPSAYPAPSSYPAPSAY

pep128 F 8 PAHSYAAPAYKPAAYKAY coat protein, virions

pep129 F 9 AAHSYSAPAYKPVAYKAY virions

pep130 F10 TPAHSYAAPAHSYAAPAH

pep131 F11 SAAPAYKQSYAAPAYKKY coat protein, virions

pep132 F12 HAAPAYKQSYAAPAYKQY coat protein, virions

pep133 F13 YSFEYSVNDPHTYDVKSQ pepS,Ap2-03

pep134 F14 PAYSPAPAAYSSSSYSTP

pep135 F15 HQHHSQVPNSYHFNYAVH

pep136 F16 NYAVHDPVTGDEKSHNEV Ap2-03

pep137 F17 SHNEVSDGHGTVKGTYSL coat protein, virions

pep138 F18 GTYSLVEPDGSIRVVEYT Ap2-05,Ap2-06

pep139 F19 VVEYTADDVHGFRAEVKK virions

pep140 F20 AEVKKIQPQHKPSSTQHT virions

pep141 F21 QHTFDLAEHKVPYFLNPT pepS virions

pep142 F22 AQAEQAHAPAPYNFEYSV

pep143 F23 GSTRVVEYIADDHSGFNA Ap2-03,Ap2-05,Ap2-06, Ap2-07

pep144 F24 GFNAEVKKIEGHIQGYKA virions

pep145 G 1 AYSAPAYSAPTYSAPAHS

pep146 G 2 PAYSASAYSAPAYSAPAY

pep147 G 3 AYSAPAHSASAYPAPAYS

pep148 G 4 VHDTYTGDIKSQNEYADA Ap2-03,Ap2-04

pep149 G 5 PAYSAPSPYNFDYSVHDT Ap2-05,Ap2-06

pep150 G 6 GSYSLVEPDGSKRTVEYT Ap2-05,Ap2-06

Name Location^†^ Sequence Anti-CuP binding^‡^ CMV binding^§^

pep152 G 8 AYPAPAYPAAPAYSAPAY

pep153 G 9 PSAPAYPSAPAYPAAPAY

pep154 G10 PAAPAYPAAPAYSAAPAY

pep155 G11 KGSYSLVEPDGTKRIVEY

pep156 G12 APAYGYSTPAPAYSAPAY

pep157 G13 PAPAYGYSTPAPAYGHAA

pep158 G14 GSTRVVEYTADNEHGSTL Ap2-01,Ap2-05,Ap2-06, Ap2-07

pep159 G15 GHGNVKGSYSLVEADGST

pep160 G16 YNGFNAEVKKVEGGYKAP

pep161 G17 DYNGFVAEVKKEGTPSYS Ap2-03,Ap2-10

pep162 G18 KPAKAAYSAPAYKPAVYS virions

pep163 G19 KAPAYPAAPAYPSAAPAY

pep164 G20 YSAPAYSAPAPAYSAPAY

pep165 G21 YTADDYSGFNAEVKKIEG

pep166 G22 AYKSAPAAYAGHSYAAPA

pep167 G23 EGHSNGYSAPAYKSAPAY virions

pep168 G24 GHGHGYSAPAYKAAPAYK coat protein, virions

pep169 H 1 GHSYAAPAHSYAAPAAYA

pep170 H 2 PAAYAGHSYAAPAAYAGH

pep171 H 3 APAAYAAHSYAAPAAYAG

pep172 H 4 AAPAAYASHSYAAPAAYA

pep173 H 5 YKPSYAAPAYKPSPYKQY coat protein, virions

pep174 H 6 APSAYPAPAYKPASYSAP

pep175 H 7 SYSAPKAYAPEAAYAPAP

pep176 H 8 PYNFDYSVHDDSTYDIKS pepS,Ap2-03

pep177 H 9 VEADGTKRIVEYTADDVN Ap2-01,Ap2-05,Ap2-06,Ap2-07

pep178 H10 ADDVNGFNAEVKKEGTPS

pep179 H11 PAPAYGYSTPAPAYKPSY Ap2-10 virions

pep180 H12 EGTPSYSSAPAYKPAYKA Ap2-10 coat protein, virions

pep181 H13 PAAPAYPTESYPAAPAYG

pep182 H14 KPAYKAPAYPAAPAYPSA

pep183 H15 HGYSAASAYRAAPAYPSY virions

pep184 H16 HSGFNADVKKEGGYAAP

pep185 H17 PAYPSYPAAPAYPAYPAA

pep186 H18 SLVDADGSKRTVDYTADD Ap2-05,Ap2-06, Ap2-07

pep187 H19 QTEYADANGYVKGTYSLV virions

pep188 H20 YSVHDSHTGDIKSQTEYA Ap2-03,Ap2-04

pep189 H21 ATAAAPSSPSPYNFQYSV

pep190 H22 AYYKPAQYSAPKPYAATA

pep191 H23 AAPAYNKPTAYNSAAAYY

pep192 H24 AYQAASAYQAAPAYHAAP

pep193 I 1 QAAPAYQAYQAAPAYQAA

pep194 I 2 PAYPAYHAAPAYSVYQAA

pep195 I 3 PAAPAYPAASAYSAAPAY

pep196 I 4 SAASAYRAAPAYPSYSAP

pep197 I 5 PAYSSHQAPAYQAHGYSA

pep198 I 6 APAYSSNQAPAYSSNQAP

pep199 I 7 HHHESLHPYEQSSYDKFY

pep200 I 8 DGHGNVKGSYRLVEPDGS

pep201 I 9 EPDGSTRVVEYTADHEHG Ap2-03,Ap2-07

pep202 I10 DHEHGFNAVVKKIDAPHY virions

Name Location^†^ Sequence Anti-CuP binding^‡^ CMV binding^§^

pep204 I12 HHDHYAHAPAPYHFEYGV Ap2-03

pep205 I13 SHVDVDYHQQPSNYHKYY coat protein, virions

pep206 I14 QHHELLHPYDHSSYDKFY Ap2-03

pep207 I15 IEAPKDNNNYYYHQSHVD

pep208 I16 PHYQGDYSAHTTDYHQQH

pep209 I17 SYDGAGHLQYQHSGYTPP

pep210 I18 GYTPPAAPYHFEYGVKDL

pep211 I19 KDLHTHDIKSQQEVSDGH Ap2-03

pep212 I20 HHHYESLHPYKHLSYNKF coat protein, virions

pep213 I21 DAPHHHVEYSNTADHHHY

pep214 I22 HHAHHPQHYGHHEHHEEE Ap2-03

pep215 I23 ENTHHDGHDHYAPPHYTY

pep216 I24 HYTYKYGVKDPHTGDHKH Ap2-03 coat protein, virions

pep217 J 1 DHKHQSEHRDGDVVHGEY

pep218 J 2 HGEYSVVEPDGRVRKVTY coat protein, virions

pep219 J 3 TYTADKHNGFNAHVHHVH

pep220 J 4 HHKHHPRHHHRRGGRQQF coat protein, virions

pep221 J 5 HVHYENHAHHPQVHHHHK coat protein

pep222 J 6 HHTQHHGHDYYAPPKYTF virions

pep223 J 7 PKYTFKYGVKDPHTKDDK virions

pep224 J 8 TKDDKHQWEERDGDVVHG

pep225 J 9 HGGYSLVEPDGRIRKVTY coat protein, virions

pep226 J10 GTSVPSGDYGHKGQNPVG

pep227 J11 VGGPSGSDGNSEPEPFNF Ap2-02,Ap2-03

pep228 J12 PFNFAYQVKDAPTNTDFK virions

pep229 J13 NTDFKHEANSDGKRVTGA

pep230 J14 RVTGAYSVLLPDGRNQVV

pep231 J15 RNQVVTYVADENGYNAKI Ap2-03

pep232 J16 PTGVKGSYAAPQPKNGGY virions

pep233 J17 AKINYEGEAKPQPSQPGS

pep234 J18 PGSQGGYPSAPGFPSAAP

pep235 J19 APSYPSAAPGYPSAAAPG

pep236 J20 APGYPSAAAPGYPSSASG

pep237 J21 ASGFPSSAPGGYPSSAPS

pep238 J22 SYPSSAPGFPSSAPSYPT

pep239 J23 GISVSSGDYGYKGQNSVG Ap2-03 virions

pep240 J24 KNGVKGSSRPKIGDFDFD Ap2-03

pep241 K 1 NAKVSYEGEAKPQPRPPG

pep242 K 2 PGIQGGYLTAQGFPATAL

pep243 K 3 ALKYPLPAYGSAPSPADY Ap2-03

pep244 K 4 DYPGSAYSAPVSGYSGFY Ap2-03

pep245 K 5 YGGSPPAPSYLVQIPTFK virions

pep246 K 6 LRYDDGTGYGPLEGYQQW

pep247 K 7 GDIKDQQETKNGDQLTGY Ap2-03

pep248 K 8 QLTGYYRLLDSDGLVRTV

pep249 K 9 LVRTVNYQSHPLTGFTAQ

pep250 K10 GFTAQVNRDPIGSEGQAK virions

pep251 K11 EGQAKYRSAEAAQQARTA

pep252 K12 TAWPSNLRAPLVPSNQYL Ap2-03

pep253 K13 RTVEYTADDYNGFNAVVK Ap2-05,Ap2-06 virions

pep254 K14 AIVHKSGHANHPDHYEHY

Name Location^†^ Sequence Anti-CuP binding^‡^ CMV binding^§^

pep256 K16 VQDPHTGDYKTAHEYRDG Ap2-03

pep257 K17 RDGDVVKGSYSVHDPDGT

pep258 K18 GTLRTVEYVADKENGFNA Ap2-03

pep259 K19 APVYAPAVSYPFPYKRQA coat protein, virions

pep260 K20 ASPVAYPAYPAAAYQPVN

pep261 K21 PVNPNPSYAYKYGVSDPA

pep262 K22 PATGDYKTAEESLSNGVV Ap2-03

pep263 K23 GVVQGQYSLAEPDGTVRT

pep264 K24 VRTVSYTADDVNGFVAQV Ap2-03,Ap2-05, Ap2-07

pep265 L 1 AQVTKGAKVIAPAAPVYS

pep266 L 2 VYSAPAPVYSAPAPVYAA

pep267 L 3 VYAAAPAPVYAPAVHAPV

pep268 L 4 PAVKAYAPSPYHTYPAYH virions

pep269 L 5 EAAPYSPTLYAPVPYYAA

pep270 L 6 YAAAAPKSYDADYDPNPS

pep271 L 7 NPSYSYAYDVHDQLTGDS Ap2-03

pep272 L 8 GDSKSQHESRHGDVVHGS Ap2-03

pep273 L 9 HGSYSLVDPDGTRRTVDY virions

pep274 L10 TVDYTADPHNGFNAVVTK virions

pep275 L11 VVTKEPLAKGYGAAPARA virions

pep276 L12 PARAAYPVKSYAPAPAVY

pep277 L13 PAVYPAAVNAYAPIYPAV

pep278 L14 HHPAQVAHAPVYHHAYRR coat protein, virions

pep279 L15 PAAIGYHHGGAPAAYHHQ

pep280 L16 HHQGYDDAQSYDYAAYPA

pep281 L17 YPAYSSASTYQADYPSYS Ap2-04

pep282 L18 SYSSKPVDYYSPPKYSFN virions

pep283 L19 SFNYGVKDYHTGDVKDQW Ap2-03

pep284 L20 DQWEERDGDVVKGEYSLV Ap2-08

pep285 L21 SLVEPDGTTRKVTYTADD Ap2-05, Ap2-07

pep286 L22 DDHNGFNAVVHKSGTAHH coat protein, virions

pep287 L23 YPLQEHQPELHKLSSSHR coat protein, virions

pep288 L24 GHYDSSTAEHSEAVGDSS Ap2-03,pepL

pep289 M 1 IEPVHRQQHDLSQSVASP pepS

pep290 M 2 ASPSYKFDFAAEHAAAAP pepS

pep291 M 3 APPQVDYELQQSLHYNYP

pep292 M 4 ISVPSYPSPSSPSAPSAP

pep293 M 5 YSAAPAYPAASAYSTPAY

pep294 M 6 KPSPSYSAPSYSAPSYSA

pep295 M 7 APSAPSAPSYSPAPSYSP

pep296 M 8 APLYSPAPSYSPAPAYKP virions

pep297 M 9 PAPAYKPAPAYKPAPSYK virions

pep298 M10 SYAAPAYSAPAPYKSQEP

pep299 M11 EPEYPAKPYSFDYSVNDY Ap2-03

pep300 M12 DYQTGDVKSQAEYSDGKN Ap2-03

pep301 M13 KNVKGYYSLIEADGTKRI virions

pep302 M14 RIVEYTADEYGFNAVVKK Ap2-03 virions

pep303 M15 KKEGTPSYAPAAPAYKAP

pep304 M16 AYPTAPAYRAAPAYPAVP Ap2-03

pep305 M17 MTSCRPSAAPFAFAPPAT

pep306 M18 QHMHFYLPEQYVITSVRR Ap2-03 coat protein, virions

Name Location^†^ Sequence Anti-CuP binding^‡^ CMV binding^§^

pep308 M20 AASIDSSDHRPSSDLGFG

pep309 M21 RLGGGGDSSVEIIRSTDQ Ap2-03

pep310 M22 ATPSGITASVAPPGYDFG Ap2-03

pep311 M23 GHPHVVDTGHQQQQQQQP

pep312 M24 QPSELDDLGHLVNQLHQL Ap2-03

pep313 N 1 QLSEHEDFSHHYENQLQL

pep314 N 2 QLQAQQASEHEDLGHNAD Ap2-03

pep315 N 3 ADDRQPSTEAPPADSSYN

pep316 N 4 NFAYSVEDPLTGDVKSQT Ap2-03

pep317 N 5 TEVSDGRGTVKGSYSMVE

pep318 N 6 SAPAYSAPKAYAPEPAYA

pep319 N 7 EPGVLPAVAPSPYAYPGL

pep320 N 8 VQKDVPVVAPAVAPAVVV

pep321 N 9 GLLPYSAAPAAYPYSAAP

pep322 N10 APGAYPYAAYPFRAPLLP

pep323 N11 PAASRAVASPLQAFPAPA

pep324 N12 PAARFAAAPFPAPAAVAP

pep325 N13 APFPAGAPLVRAAAPLPV

pep326 N14 PVAAAPLPVAAAPAVAKL

pep327 N15 KLDFTDAYPQYQFAYTVR virions

pep328 N16 VRDSLTGDAKDQEEVRDG Ap2-03

pep329 N17 DGDVVKGRYSLIEPDGSR virions

pep330 N18 RRTVNYYADDVNGFNAVV

pep331 N19 AVIDEPSAPAQYDFSYAV

pep332 N20 SYSQYAYNGQYGSPVVRK coat protein, virions

pep333 N21 AVNDPTTGDQKDQQESRN

pep334 N22 RNGDDVTGYYRTLDSDGY

pep335 N23 GYLRTVKYKADAVNGFTA

pep336 N24 TAEVVREPVSAAAAAVPV

pep337 O 1 PVVAKAAAVIKPLVAPVV virions

pep338 O 2 VVPAVAPVAPVVPYVAPV

pep339 O 3 PVPYVASPAPFYFGSSGY

pep340 O 4 GYPYNYQPYSYGGYQQYP

pep341 O 5 YPYNAGYPYNAGYSPYSY pepL buffer

pep342 O 6 QYGRPAGNSNPGYGGTGT Ap2-03

pep343 O 7 PALAPASGPSSAPSLVKY virions

pep344 O 8 GTSVPSGYYEQKGQYPGS

pep345 O 9 GSTGTNIPSGYYGQKGQY coat protein, virions

pep346 O10 QYPVVQVPIYHQILWTKS virions

pep347 O11 KSQYPVETCGSDGNSEPE Ap2-02

pep348 O12 PEPFNFAYQVKDAPTNTY

pep349 O13 TYFTHEANSDGKRVTGAY virions

pep350 O14 AYSVLLPDGRNQVVTYFA

pep351 O15 FADENGYNAKVSYEGEAK

pep352 O16 AKPQPAQPGSQGGYLSAP

pep353 O17 APGFSSTAPKYPPVPIRG virions

pep354 O18 RGSAPRPTGYPGSAIPAS

pep355 O19 ASAPAPGYSSSAPTFGSP Ap2-03

pep356 O20 LDCSPSYSSGYEQKEYSS Ap2-03,Ap2-04

pep357 O21 SSYGAPAKQSQEYEAPSY

pep358 O22 SSHSAPSYGPKPQAYSAP virions

Name Location^†^ Sequence Anti-CuP binding^‡^ CMV binding^§^

pep360 O24 APSYSAPSYSSPAYGKPQ virions

pep361 P 1 PQAYSYPSSYSAPSYSAP

pep362 P 2 APSHSAPSYSAPSHSAPS

pep363 P 3 SAPSHSALSYSAPSYSAP

pep364 P 4 APSHYSPSHSASSYSPAS

pep365 P 5 ASQAYGKPQSYSAPSYNA virions

pep366 P 6 NAQSYSAPSYSTPAYGKQ virions

pep367 P 7 KQQSYSAPSYSAPSYSAP

pep368 P 8 APAYGKQQSYSAPSASY virions

pep369 P 9 SYEQPKYPVSQYSAPSYE

pep370 P10 YEAKPQSYSAPTPSYNKE

pep371 P11 KEPSHSASAYPSYPSQKA virions

pep372 P12 KAHAEESYAPKPYKFEYS

pep373 P13 YSVNDEHTYDIKSQKEES pepS,Ap2-03

pep374 P14 ESDGYHVKGYYTLLEADG

pep375 P15 DGSRRTVEYTADENGFNA Ap2-03,Ap2-05,Ap2-06, Ap2-07

pep376 P16 NADVKKEEAQGYNKAAPA Ap2-09

pep377 P17 PAYNKDSQAYKSAPAAYS virions

pep378 P18 AAYSAPSYNTQVSPYGAQ

pep379 P19 GAQPSYNAPAYNKQESSY

pep380 P20 PSSYSSSSYSSSSYSTAA

pep381 P21 AASYSSASYSAPSYSAQS

pep382 P22 SYAPAAPSYAPAAPSYAP

pep383 P23 SQSYQSASYSAAAPSYPS

pep384 P24 SPSYAPAPAPAYAPAAQS

^†^Location of the peptide on the printed array with the column (letter) and row (number) indicated for each peptide.

^‡^Name of the anti-RR-2 antibodies which hybridized significantly (intensity ≥8,000) for each peptide on the array

^§^Virions of *Cucumber mosaic virus* (vir. CMV), or CMV coat protein (CMV-CP), which hybridized significantly (intensity ≥8,000) to peptides on the array.
